# Raising awareness and mitigating risk of transmission of antimicrobial resistance during the upcoming 2024 Gangasagar religious mass gathering

**DOI:** 10.1016/j.nmni.2023.101213

**Published:** 2023-12-19

**Authors:** Avinash Sharma, Bhavuk Gupta, Abhrajyoti Ghosh, Shilpi Sharma, Alfonso J. Rodriguez-Morales, Alimuddin Zumla, Ziad A. Memish

**Affiliations:** DBT-National Centre for Cell Science, Complex, Ganeshkhind, Pune 411007, India; School of Agriculture, Graphic Era Hill University, Dehradun, Uttarakhand, India; DBT-National Centre for Cell Science, Complex, Ganeshkhind, Pune 411007, India; Department of Biological Sciences, Bose Institute, Kolkata, India; Department of Biochemical Engineering and Biotechnology, Indian Institute of Technology Delhi, New Delhi, 110016, India; Universidad Científica del Sur, Lima, Peru; Grupo de Investigación Biomedicina, Fundación Universitaria Autónoma de las Américas, Risaralda, Colombia; Department of Infection, Division of Infection and Immunity, University College London; and NIHR Biomedical Research Centre, UCL Hospitals NHS Foundation Trust, London, UK; Research and Innovation Center, King Saud Medical City, Ministry of Health & College of Medicine, Al Faisal University, Riyadh, Saudi Arabia; Hubert Department of Global Health, Rollins School of Public Health, Emory, University, Atlanta, USA

**Keywords:** Mass gatherings, Religious event, Antimicrobial resistance, Gangasagar, AMR awareness

Compared to the Kumbh Mela which attracts over 50 million pilgrims, the Gangasagar Mela (GSM) is India's second largest religious mass gathering event (MGE). The GSM, is an annual event held in Gangasagar West Bengal, the Kumbh takes places every three years, rotating among four different Indian cities, viz. Nashik, Ujjain, Prayagraj and Haridwar. The MGEs frequently result in significant and repeated human-induced disruptions at the event site, causing adverse impacts on the local ecosystems. Annually, during the month of January, GSM witnesses the participation of millions of pilgrims over a duration of a few days bathing and living together performing religious rituals. In addition to this annual event, Gangasagar is continuously stressed by the discharge of industrial and agricultural effluents upstream.

Large-scale mass gatherings, such as religious pilgrimages, and sports events, attract multitudes of participants from diverse geographic and cultural backgrounds [[1], [2], [3], [4], [5]]. Despite fostering communal bonds and celebrations, the WHO highlights that such mass gathering events are associated with serious health risks due to disease transmission, because of the close interactions among attendees. As a consequence, equally critical is the looming threat of development and spread of antimicrobial resistance (AMR) during and after MGEs. Due to the increased human-human interactions and the potential for disease transmission, the setting of MGEs are hot spots for the proliferation of AMR. Apart from the human to human transmission of a range of pathogens at MGEs, these events impose stress on the temporary healthcare facilities established for the event, impacting native animal species and ecosystems, and contributing to the spread of AMR genes within the waste water infrastructures, thereby posing challenges to efficient waste management (Fig. 1) [[1], [2], [3]]. Despite these environmental and health concerns, the major gap in the context of GSM is the lack of thorough surveillance of AMR, and identification of risk factors contributing to development and dissemination of AMR. In the absence of efficient waste management approaches, the huge amount of waste generated over a couple of days due to such congregation, contributes to the transmission of AMR to natural environments [[6], [7], [8], [9]]. Also, the abundance of AMR has been reported to increase under the influence of anthropogenic factors, including the presence of pollutants, especially the emerging contaminants [10]. While natural attenuation has been observed for Gangasagar, the deterioration of soil and water quality post GSM has been documented [8]. It is worth noting that being the largest consumer of antibiotics globally, India is a victim to rising cases of AMR, as evidenced by the 2022 annual report from the Indian Council of Medical Research “Antimicrobial Resistance Surveillance Network”, which depicts a growing trend in the emergence of AMR pathogens [9], [10], [11], [12].

**Fig. 1:**
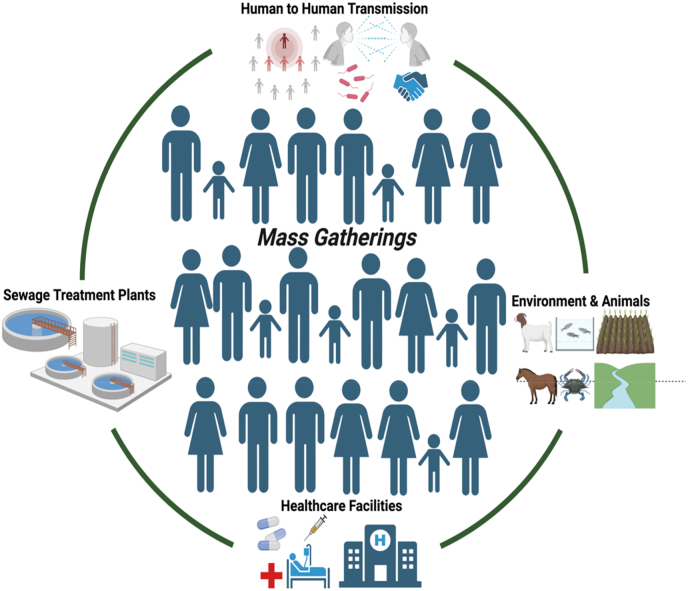
Illustrates potential areas of AMR spread during mass gatherings.

The emergence of the COVID-19 pandemic in December 2019 serves as a significant scientific lesson, underscoring the imperative of implementing rigorous global policy measures. This crisis prompted the enforcement of extensive lockdown measures worldwide to mitigate its transmission. However, this pandemic disrupted proper antibiotic management, leading to a marked increase in the worldwide administration of antibiotics and biocides. Consequently, this has exacerbated the overarching problem of development and transmission of AMR globally, while simultaneously augmenting the occurrence of emerging contaminants within water ecosystems. It is important to note that deaths due to COVID-19 worldwide was over 4 million, however AMR contributed to an estimated approximate deaths of 4.95 million globally in 2019 alone [6]. The death toll from AMR is a cause for concern, with its silent and less-recognized pandemic status. If these numbers continue to rise, AMR will pose a significant and underappreciated threat to global public health, potentially overshadowing the impact of COVID-19.

To mitigate the development and transmission of AMR during the upcoming GSM, it involves adopting strategies before, during, and after the bathing event. This necessitates a collective effort from all stakeholders and comprehensive data collection and surveillance at each stage to assess the gravity of the situation. WHO's Global Antimicrobial Resistance Surveillance System (GLASS) is pivotal in addressing this issue by collecting and sharing data at local, national, and global levels for informed decision-making and combatting antibiotic resistance, as it can establish monitoring mechanisms for early detection of potential outbreaks and tracking patterns related to AMR and infections during such large-scale events.

Besides developing a strong infrastructure for healthcare, hygiene and sanitation, the preparedness for the event must also strengthen the public health system in advance. Additionally, prioritizing education about appropriate antibiotic usage, promoting responsible behaviour, and discouraging self-medication are crucial strategies for public health awareness and tackling the fast-growing issue of AMR. [[13], [14], [15]]. Several conventional and advanced platforms can be utilised for such an awareness, including reinforcement by religious and community leaders. A pre-registration of pilgrims and visitors with documentation of any illness history and or medicament, can be a robust method of monitoring.

During the event, health authorities should actively monitor and regulate antibiotic dispensation, implementing measures like requirement of prescriptions for controlling antibiotic sales, and providing healthcare professionals at gathering sites to evaluate and prescribe medications as needed. Availability of point-of-care devices to rapidly detect infections can curb the overuse (and unnecessary use) of antibiotics, with more precise treatment. Visitors and pilgrims should be required to report signs of illness promptly to designated healthcare facilities. Additionally, these events generate substantial human waste, placing significant stress on effective waste and sewage management systems, to minimize transmission of AMR to other ecosystems. This highlights the need for sewage-based AMR surveillance to address this multifaceted issue [15], [16], [17], [18]. Post the event, the pollution and waste generated by the gathering, must be sustainably managed to minimize the transmission of pathogens and AMR to water and soil. Further, AMR pathogens studied during mass gathering events should be archived in microbial repositories 19, along with their associated data, for the benefit of future research and to enrich our understanding of the course of AMR spread, in collaboration with the Indian Council of Medical Research (ICMR).

The One Health approach [20], [21] is crucial in addressing the interconnected challenges of AMR, infectious diseases, and public health during mass gatherings. These limitations underscore the need for a comprehensive and authentic database to bridge these critical knowledge gaps in the battle against antimicrobial resistance. The Government must continue supporting research in new therapeutics, together with investing in long-term monitoring across seasons for the dynamics of pathogens and AMR. This will enable a better understanding of the resilience of the system so that appropriate and timely interventions can be undertaken.

## Author declarations

All authors declare no conflicts of interest. The views expressed are those of the authors and not necessarily those of their respective institutions.

## CRediT authorship contribution statement

**Avinash Sharma:** Conceptualization, Writing – original draft, Writing – review & editing. **Bhavuk Gupta:** Writing – review & editing. **Abhrajyoti Ghosh:** Writing – review & editing. **Shilpi Sharma:** Writing – original draft, Writing – review & editing. **Alfonso J. Rodriguez-Morales:** Writing – review & editing. **Alimuddin Zumla:** Conceptualization, Writing – original draft, Writing – review & editing. **Ziad A. Memish:** Writing – original draft, Writing – review & editing.
